# Notes and News

**Published:** 1905-05-13

**Authors:** 


### NOTES AND NEWS.

THE Royal Waterloo Hospital for Children and Women is asking for volunteer workers to assist in collecting funds for the hospital; any offers of help should be addressed to the Secretary at the hospital.

W. J. LANCASTER, Esq., J.P. (ex-Mayor of Wandsworth), will preside at the annual dinner of the Royal Hospital for Incurables, which will be held at the Savoy Hotel, Strand, on Friday, June 30th, at 6.30 for 7 P.M.

DR. W. PAGE MAY, Fellow of University College, will give ten lectures on consecutive Wednesdays, commencing June 5th, at 5 P.M., on "The Structure and Functions of the Central Nervous System." These lectures are open to medical practitioners on presentation of their cards.

THE Summer Course of Post-Graduate Lectures and Demonstrations in connection with Mount Vernon Hospital for Consumption and Diseases of the Chest, Hampstead and Northwood, will be held at the Central Out-Patient Department, 7 Fitzroy Square, W., on Thursdays, at 5 P.M. Members of the profession and medical students are invited, and will be admitted on presentation of their address cards.

THE following is the programme of lectures. May 18, Dr. George A. Heron on "Some of the Problems to be Solved in our Efforts to Eradicate Tuberculosis." May 25, Dr. J. Edward Squire, on "Difficulties in the Diagnosis of Chest Affections." June 1, Dr. George Johnston on "The Pathology and Treatment of Asthma." June 8, Dr. F. Parkes Weber on "Pneumothorax in Tuberculous Subjects." June 15, Dr. T. N. Kelynack on "Demonstration of Cases of Arrested and Advancing Pulmonary Tuberculosis." June 22, Dr. T. D. Lister on "Pre-Tuberculous Affections in Early Life." June 29, Dr. F. W. Price on "Aortic Stenosis." July 6, Mr. James Berry on "Foreign Bodies in the Air-passages." July 13, Mr. Harold Barwell on "The Diagnosis of Tuberculous Laryngitis in its Early Stage." July 20, Dr. Herbert Scharlieb on "Some Post-Anæsthetic Complications." As far as possible the lectures will be illustrated by cases.

A NEW cottage hospital has been opened at Ilkley. It is for nine surgical cases, and was established at a cost of £2,500.

THE London Congress of the Royal Institute of Public Health will be held from Wednesday, July 19, to Tuesday, July 25.

THE Lord Mayor will preside at a meeting to be held at the Mansion House on May 17, at 3 o'clock, in aid of the National Association for the Prevention of Consumption, which is appealing for £10,000.

THE Lord Mayor will preside at a meeting to be held at the Mansion House on July 13 to commemorate the 21st anniversary of the establishment of the Mary Wardell Convalescent Home at Stanmore.

A NEW wing has been added to the Scarborough Cottage Hospital. This adds two wards with eight beds in each, and other accommodation, the cost of providing which has been £1,000. This sum was provided through a legacy from the late Mr. Daniel, of York. The architect was Mr. Caleb Petch.

A FINE institution for the reformation of inebriates has been opened at Cattal, in Yorkshire. It is the outcome of a conference representative of the three Ridings of Yorkshire in 1899. The Home commences with accommodation for about 100 patients, some of whom will probably be voluntary paying patients under the Act of 1879. The buildings consist of a large main block and separate cottage homes, which will admit of careful classification. There are about 51 acres of land purchased for £4,750, and the building and furnishing cost about £50,000.

WE recently published a graphic account of the Lunatic Department in a French hospital. The description is a sad one, and reveals the deplorable condition of the unfortunate patients. This description is quoted without comment in the *Guernsey Advertiser*. Does the editor intend the inhabitants of Guernsey to draw attention to the treatment of the insane in Guernsey by this picture in the French hospital, in the hope that the lesson will go home? Unfortunately the Guernsey people have not far to seek a parallel which forms an equal blot on their humanity and their progress in scientific methods.

DR. NEVINS, of Liverpool, in a recent address on the subject of consumption sanatoria, remarked that if the notification of consumption became compulsory he feared more harm than good would result, as it would tend to the concealment of the disease. If the regulations are judiciously drawn up we cannot see why this should be the case. Notification in the case of consumption would hardly tend as a necessary sequence to segregation in sanatoria, seeing that medical opinion generally is becoming to accord with Dr. Nevins' view that the treatment is very far from inducing a certain cure. But notification might usefully be followed up by judicious supervision and education in those precautions which tend to safeguard the public from infection.

